# Chronic Acral Lesions (“COVID Toes”): To Add to Long Post- COVID-19 Syndrome?

**DOI:** 10.1177/00033197211068938

**Published:** 2022-01-03

**Authors:** Raphaël André, Aurélie Hsieh, Laurence T. Trellu

**Affiliations:** 1 Department of Dermatology, 27230 Geneva University Hospitals, Geneva, Switzerland

Dear Editor,

**“**COVID toes” are known to be associated with severe acute respiratory syndrome coronavirus (SARS-CoV-2) pathogenesis.^1^ Descriptions are concordant worldwide, however, follow-up is poorly described. Between February 2020 and April 2021, 4 cases lasting >3 months were followed-up in our dermatology clinic.

**Figure 1.**
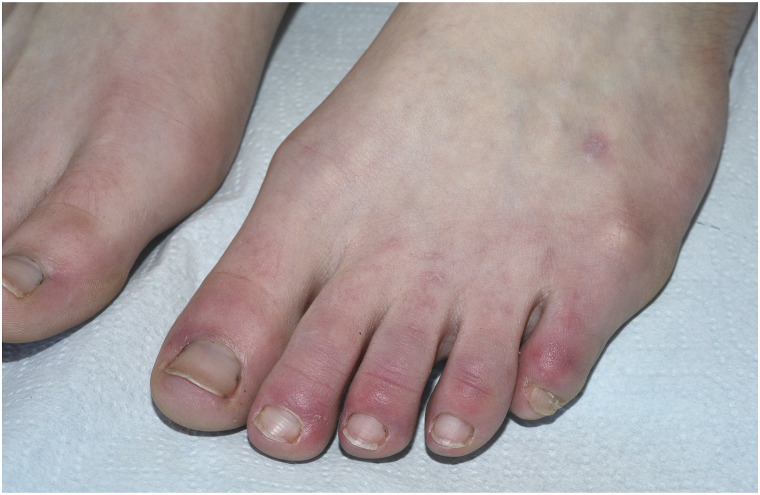
Chilblain-like acral lesion on left foot.

The first patient was a 19-year-old woman without medical history. Two months after the onset of COVID-19 symptoms, chilblains-like acral lesions (CLAL) appeared on her toes (Figure 1). She complained of itching. There was no worsening with cold. SARS-CoV-2 serology was positive, and antinuclear antibodies, cryoglobulin, cryofibrinogen, cold agglutinin were negative. Chilblains-like acral lesions were still visible 4 months after onset. The second patient was an 18-year-old woman in good health. Two weeks after the onset of COVID-19, asymptomatic CLAL appeared on her toes and fingers. SARS-Cov-2 infection was confirmed by polymerase chain reaction (PCR). Moreover, she presented with a transient and painless urticarial eruption of the trunk, knees and hands exacerbated by heat and disappearing with cold. We found positive antinuclear antibodies with nucleolar fluorescence and rheumatoid factor, but there was no clinical systemic lupus sign. CLAL were still visible at 3 months. The third patient was a 60-year-old woman in good health. Soon after the onset of a COVID-19 with mild typical symptoms (anosmia), she presented painless CLAL of her toes. She did not performed any viral test. She noted an exacerbation by heat, but no worsening by cold. One digital necrosis was observed and CLAL remained for 5 months. The fourth case was a 20 year-old female who presented symptoms consistent with COVID-19 infection, but with a negative serology. Two months later, CLAL and erythromelalgia appeared on her toes and persisted after 10 months showing superficial necrotic evolution.

Our case series shows a CLAL duration of 3 to 10 months. Symptoms vary from no pain to functional disability. Cold was not a worsening factor, which differs from idiopathic chilblains. Necrotic evolution is possible as shown in 2 cases. Based on recent data, CLAL reflect an immunity against the virus by secretion of type 1 alpha-interferon.^2,3^ Indeed, few patients with COVID toes have developed a severe form of the virus,^1^ and serologic or PCR test for SARS-Cov2 are sometimes negative.^4^ Among our 4 patients, 2 were positive, 1 negative and 1 not performed. Our data show a long duration of these symptoms but CLAL have not been integrated in the concept of “long COVID” (ie persistent complications),^5^ no pediatric acral acute syndrome had this duration.^6^ We suggest to add acute and chronic COVID toes as a separate post-infection entity.
